# Fatigue Damage Evaluation Using Nonlinear Lamb Waves with Quasi Phase-Velocity Matching at Low Frequency

**DOI:** 10.3390/ma11101920

**Published:** 2018-10-09

**Authors:** Wujun Zhu, Yanxun Xiang, Chang-jun Liu, Mingxi Deng, Congyun Ma, Fu-zhen Xuan

**Affiliations:** 1 Key Laboratory of Pressure Systems and Safety of MOE, School of Mechanical and Power Engineering, East China University of Science and Technology, Shanghai 200237, China; zhuwujun20@126.com (W.Z.); cjliu@ecust.edu.cn (C.-j.L.); macongyun@foxmail.com (C.M.); fzxuan@ecust.edu.cn (F.-z.X.); 2 College of Aerospace Engineering, Chongqing University, Chongqing 400044, China; dengmx65@yahoo.com

**Keywords:** ultrasonic nonlinearity, fatigue damage, lamb wave, low frequency

## Abstract

Due to the dispersive and multimode natures, only nonlinear Lamb waves with exact phase-velocity matching were generally used in previous studies to evaluate the evenly distributed microstructural evolution in the incipient stage of material degradation, because of the cumulative generation of second harmonics, which was also found within a significant propagation distance for mode pair S0-s0 with quasi phase-velocity matching at low frequency. To explore the feasibility of fatigue damage evaluation by using this mode pair and fully utilize its unique merits, the cumulative second harmonic analysis was performed on aluminum alloy specimens with various material damage produced by the continuous low cycle fatigue tests. Similar to mode pair S1-s2 with exact phase-velocity matching, a mountain shape curve between the normalized acoustic nonlinearity parameter and the fatigue life was also achieved with the peak point at about 0.65 fatigue life for mode pair S0-s0, even though a relatively higher sensitivity to fatigue damage was observed for mode pair S1-s2. The excited frequency selection was further analyzed in a certain frequency range, where the quasi phase-velocity matching condition was satisfied for mode pair S0-s0 owing to the less dispersive property. Results show that the fatigue damage can be effectively detected using the mode pair S0-s0, and a relatively lower excited frequency was preferred due to its higher sensitivity to microstructural evolution.

## 1. Introduction

Nonlinear ultrasound has been found to be a useful indicator of the material damage within large plate-like structures for nondestructive evaluation and structural health monitoring, since they are sensitive to microstructural evolution at the early stage of material degradation when compared with the conventional linear ultrasounds [1,2,3,4,5,6,7,8,9,10,11,12,13,14,15,16,17,18,19,20,21,22,23]. The long propagation distance and the interrogation of the entire thickness of plate-like structures make nonlinear Lamb wave a highly efficient inspection method. The discipline of nonlinear Lamb wave generally consists of higher harmonic generation, sub-harmonic generation, mixed-frequency wave, and nonlinear resonance, etc. [1,2]. Recently, many studies focused on the second harmonic generation (SHG) to characterize the material nonlinearity [3,4,5,6,7,8,9,10,11,12,13,14,15]. Due to the dispersive and multimode natures, the propagation of Lamb waves is complex and the double frequency Lamb waves (DFLWs) are generally weak. Attention is usually focused on the Lamb mode pairs with phase-velocity matching and non-zero energy flux, which are demonstrated to be necessary conditions for the cumulative generation of second harmonic Lame waves [3,4,5,6,7,8,9]. Based on the second-order perturbation approximation and modal analyses approach, the amplitudes of DFLWs oscillate along the propagation distance for the mode pairs with phase-velocity mismatching [3,4,5]. Growth merely occurs to the DFLWs within half the oscillation spatial period, which depends on the deviation of the phase velocities between the primary and second harmonic Lamb waves. For mode pairs with quasi phase-velocity matching and non-zero energy flux, due to the slight deviation of phase velocities, the amplitudes of DFLWs increase cumulatively in a significant distance satisfying the measurement requirements for nondestructive evaluation and structural health monitoring.

In practical applications, the exact phase-velocity matching condition is generally difficult to satisfy due to the finite duration of the propagating tone bursts and the uncertainty in the material properties. A finite bandwidth occurs to the propagating tone bursts with the central frequency merely satisfying the exact phase-velocity matching condition [16,17]. Meanwhile, the calculated dispersive characterization according to the uncertain material properties inevitably deviates from the precise one, which leads to the slight deviation of the phase velocities between the primary and second harmonic Lamb waves for the chosen mode pairs. Consequently, the quasi phase-velocity matching condition is actually satisfied in practice for mode pairs chosen to evaluate the material degradation. Matsuda et al. explored the frequency dependence of SHG in a neighborhood of the exact phase-velocity matching frequency and revealed that the maximum amplitude of SHG occurs at the frequency close but not equal to the exact phase-velocity matching one, i.e., the quasi phase-velocity matching frequency [18]. As a typical mode pair satisfying this condition, the symmetric Lamb mode pair S0-s0 at low frequency possesses unique merits. Owing to its less dispersive property, the propagation of the nonlinear Lamb waves is more robust. Since the quasi phase-velocity matching condition is satisfied in a certain frequency range, the complicated dispersive characterization and frequency selection may be unnecessary in practical applications. Furthermore, the singular generation of the target Lamb mode occurs with other unintended modes suppressed as only S0 and A0 modes co-exist before the cut-off frequency. Also, compared with the exact phase-velocity matching based nonlinear Lamb waves, the relatively small signal attenuation owing to the low frequency makes mode pair S0-s0 suitable for damage detection in a large propagation distance. Some attempts have been made to characterize the nonlinear propagation of the low frequency Lamb waves. Chillara et al. studied the second harmonics originated from the material and geometric nonlinearities for the primary S0 and A0 modes, respectively [19]. Zhu et al. analyzed the oscillation of the acoustic nonlinearity and manifested the symmetry properties of SHG with the propagation of the primary A0 mode [20]. Wan et al. numerically verified the effectiveness of the mode pair S0-s0 for characterizing the material nonlinearity [16]. Zuo et al. experimentally and numerically validated the cumulative generation of second harmonic S0 mode along a significant distance [17]. Based on the illustrated cumulative SHG, the low frequency mode pair S0-s0 may also be used to quantitatively evaluate the early-stage material damage, similar to the nonlinear Lamb waves with exact phase-velocity matching.

Recently, many researches have been conducted to assess the fatigue damage attributed to the microstructural evolution under cyclic loading conditions using nonlinear ultrasounds, since fatigue damage significantly weakens the structural integrity and ultimately lead to the cracking and catastrophic failure [24,25,26,27,28,29,30,31,32]. While most experimental measurements were based on the nonlinear bulk waves [24,25,26,27,28,29], few studies have focused on the nonlinear Lamb waves to characterize the fatigue damage in metal materials [30,31,32]. Deng et al. reported the fatigue damage evaluation using Lamb mode pair A2-s4 in an aluminum sheet, where the monotonic decrease of the stress wave factor was observed with the number of fatigue cycles under tension-tension loading [30]. Pruell et al. chose the Lamb mode pair S1-s2 to quantitatively assessed the plasticity driven fatigue damage in Al-1100-H14 specimens with the normalized acoustic nonlinearity parameter, which was related to the cumulative plastic strain [31]. Zhu et al. validated the feasibility of fatigue damage evaluation using the Lamb mode pair S3-s6 with group-velocity mismatching owing to its high efficiency of cumulative second harmonic generation [32]. However, only the nonlinear Lamb waves satisfying the exact phase-velocity matching condition were investigated to detect the fatigue damage in previous studies as yet to the knowledge of the authors.

Therefore, the objective of the present work is to explore the feasibility of fatigue damage evaluation using nonlinear Lamb waves with quasi phase-velocity matching in the low frequency region, namely, the mode pair S0-s0, which was rarely exploited for the experimental characterization of evenly distributed material nonlinearity induced by the microstructural evolution. Firstly, the aluminum alloy plate specimens were subjected to low cyclic tension-tension loading, and the fatigue damage produced was experimentally evaluated using the Lamb mode pair S0-s0 at low frequency. The sensitivity to fatigue damage was further analyzed by the comparison between the low frequency mode pair S0-s0 and the mode pair S1-s2 with exact phase-velocity matching, which was generally used to evaluate the material damage in previous researches. To validate the experimental investigation, finite element (FE) simulations were conducted to provide a valuable insight into the Lamb wave propagation in solid plates with various levels of evenly distributed fatigue damage. Both results corroborate that the low frequency mode pair S0-s0 may be a suitable candidate for quantitatively evaluating the cyclic plasticity and hysteresis driven fatigue damage at the early stage of material degradation, even though this mode pair is relatively less sensitive to the fatigue damage than the mode pair S1-s2 with exact phase-velocity matching. Since the quasi phase-velocity matching condition was satisfied in a certain frequency range owing to the less dispersive property, the fatigue damage was further characterized using the mode pair S0-s0 at the excited frequency of 300 kHz, 350 kHz and 400 kHz, respectively, aiming to analyze the effect of excited frequency on the fatigue damage evaluation. The mode pair S0-s0 at a lower frequency was validated to be more sensitive to the fatigue damage.

## 2. Theoretical Considerations

Considering an isotropic and homogeneous solid, the nonlinear equations of motion are given by [33]

(1) $$\rho_{0}\frac{\partial^{2}U}{\partial t^{2}}=\nabla \cdot P,$$

where $\rho_{0}$ is the initial density of the solid, U the vector denoting the displacement of the particle, and P the first Piola–Kirchhoff stress tensor defined as

(2) $$P=\mu \left[\nabla U+{\left(\nabla U\right)}^{T}\right]+(\kappa -\frac{2}{3}\mu )(\nabla \cdot U)I+P^{\left(\mathrm{NL}\right)},$$

with nonlinear term

(3) $$\begin{array}{cc} P^{\left(\mathrm{NL}\right)} & =(\mu +\frac{A}{4})\left(\nabla U\cdot {\left(\nabla U\right)}^{T}+{\left(\nabla U\right)}^{T}\cdot \nabla U+\nabla U\cdot \nabla U\right)\\ & +\frac{1}{2}(\kappa -\frac{2}{3}\mu +B)\left(\left(\nabla U:{\left(\nabla U\right)}^{T}\right)I+2\nabla U(\nabla \cdot U)\right)\\ & +\frac{A}{4}\nabla U\cdot \nabla U+\frac{B}{2}\left(\nabla U:{\left(\nabla U\right)}^{T}I+2\nabla U(\nabla \cdot U)\right)+C{\left(\nabla \cdot U\right)}^{2}I \end{array}$$

Here “:” denotes the dyadic operation, κ and μ are the second-order elastic constants, and *A*, *B*, and *C* are the third-order elastic constants of the material. Substituting Equations (2) and (3) into Equation (1) yields

(4) $$\rho_{0}\frac{\partial^{2}U}{\partial t^{2}}-(\kappa +\frac{4\mu }{3})\nabla (\nabla \cdot U)+\mu \nabla \times (\nabla \times U)=F(U),$$

and

(5) $$\begin{array}{cc} F(U) & =(\mu +\frac{A}{4})\left(\nabla U\cdot \nabla^{2}U+\nabla^{2}U\cdot \nabla U+2\nabla U:\nabla \nabla U\right)+(\kappa +\frac{2}{3}\mu +B)\nabla^{2}U(\nabla \cdot U)\\ & +(\kappa +\frac{1}{3}\mu +\frac{A}{4}+B)\left(\frac{1}{2}\nabla \left(\nabla U:{\left(\nabla U\right)}^{T}\right)+\nabla (\nabla \cdot U)\cdot \nabla U\right)\\ & +(\frac{A}{4}+B)\left(\nabla U\cdot \nabla (\nabla \cdot U)+\nabla \nabla U:\nabla U\right)+(B+2C)\nabla (\nabla \cdot U)(\nabla \cdot U) \end{array}$$

where F(U) denotes the driving force. This nonlinear problem is usually solved using a perturbation approach. To consider the harmonics up to second order, U is expressed as

(6) $$U=U^{\left(1\right)}+U^{\left(2\right)},$$

where $U^{\left(1\right)}$ and $U^{\left(2\right)}$ denote the displacement vectors of the primary Lamb waves and the DFLWs, respectively. Assuming $U^{\left(2\right)}$ is very small compared with $U^{\left(1\right)}$, substituting Equation (6) into Equations (4) and (5) yields

(7) $$\rho_{0}\frac{\partial^{2}U^{\left(1\right)}}{\partial t^{2}}-(\kappa +\frac{4\mu }{3})\nabla (\nabla \cdot U^{\left(1\right)})+\mu \nabla \times (\nabla \times U^{\left(1\right)})=0,$$

and

(8) $$\rho_{0}\frac{\partial^{2}U^{\left(2\right)}}{\partial t^{2}}-(\kappa +\frac{4\mu }{3})\nabla (\nabla \cdot U^{\left(2\right)})+\mu \nabla \times (\nabla \times U^{\left(2\right)})=F(U^{\left(1\right)}),$$

where $F(U^{\left(1\right)})$ is a time-domain volume driving force in the plate, computed from F(U) using $U^{\left(1\right)}$ instead of U. There are also time-domain surface forces, $P(U^{\left(1\right)})$, acting on the surfaces of the plate.

Based on the second order perturbation approximation and modal analyses approach, the second-harmonic field of Lamb wave, $U^{\left(2\omega \right)}(y,z)$, in an isotropic and homogeneous plate without attenuation and dispersion can be expressed as [33]

(9) $$U_{}^{\left(2\omega \right)}(y,z)=\sum_{n}a_{n}(z)U_{\left(n\right)}^{\left(2\omega \right)}(y),$$

where $U_{\left(n\right)}^{\left(2\omega \right)}(y)$ is the field function and the corresponding expansion coefficient of the nth DFLW, $a_{n}(z)$, is acquired from [3,4,5]

(10) $$4P_{nn}(\frac{\partial }{\partial z}-jk_{\left(n\right)}^{\left(2\right)})a_{n}(z)=f_{Vn}(z)+f_{Sn}(z),$$

where

(11) Pnn=Re∫−d+d[−12∂∂tU(n)(2ω)⋅Tn⋅z^]dy,

(12) $$f_{Vn}(z)=\int_{-d}^{+d}j2\omega {\tilde{U}}_{\left(n\right)}^{\left(2\omega \right)}\cdot F(U^{\left(1\right)})dy,$$

(13) $$f_{Sn}(z)=j2\omega {\tilde{U}}_{\left(n\right)}^{\left(2\omega \right)}\cdot P(U^{\left(1\right)})\cdot {\hat{y}|}_{y=-d}^{y=+d}.$$

The symbols “^” and “~” denote a unit vector and complex conjugate; $T_{n}$ is the stress tensor of the *n*th second-harmonic wave component $U_{\left(n\right)}^{\left(2\omega \right)}(y)$, $P_{nn}$ represents the average power flux per unit width, $f_{Vn}(z)$ and $f_{Sn}(z)$ represent the power flux through the volume and the surfaces of the solid plate. Assuming the Lamb wave is transmitted at location z = 0, the amplitude can be expressed as

(14) $$a_{n}(z)=\frac{f_{Vn}(z)+f_{Sn}(z)}{4P_{nn}}\cdot \frac{c_{p}^{\omega }c_{p}^{2\omega }}{\omega \left(c_{p}^{2\omega }-c_{p}^{\omega }\right)}\cdot \sin\left(\frac{\omega \left(c_{p}^{2\omega }-c_{p}^{\omega }\right)}{c_{p}^{\omega }c_{p}^{2\omega }}z\right),$$

For mode pairs with phase-velocity matching ($c_{p}^{\omega }=c_{p}^{2\omega }$) and non-zero energy flux ($\left[f_{Vn}(z)+f_{Sn}(z)\right]/P_{nn}\neq 0$), $a_{n}(z)$ increases linearly with the propagation distance *z*. For mode pairs with phase-velocity mismatching ($c_{p}^{\omega }\neq c_{p}^{2\omega }$) and non-zero energy flux ($\left[f_{Vn}(z)+f_{Sn}(z)\right]/P_{nn}\neq 0$), $a_{n}(z)$ oscillates with *z* in the form of a sine function and the magnitude of $a_{n}(z)$ grows cumulatively within half the oscillation spatial period, $z_{n}$, which can expressed as

(15) $$z_{n}=\frac{\pi }{2\omega }\cdot \frac{c_{p}^{\omega }c_{p}^{2\omega }}{\left|c_{p}^{2\omega }-c_{p}^{\omega }\right|},$$

where the maximum magnitude occurs. It can be found that $z_{n}$ depends on the angular velocity and the phase velocities of the primary and second harmonic waves. When the quasi phase-velocity matching ($c_{p}^{\omega }\approx c_{p}^{2\omega }$) condition is satisfied, the propagation distance, within which the amplitude of the *n*th DFLW grows cumulatively, may be large enough for material damage evaluation in practical applications.

## 3. Experimental Details

### 3.1. Low Cycle Fatigue Test

**Figure 1:**
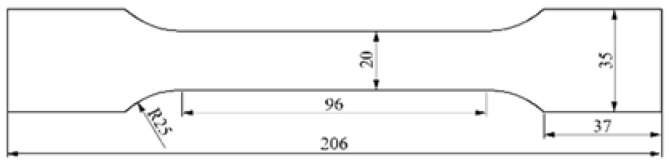
Nominal dimensions of flat dog-bone specimen. All dimensions are in mm.

To simulate the cyclic material response, the low cycle fatigue tests were conducted on the Al7075 aluminum alloy specimens at the room temperature under laboratory environment through a 500 kN servohydraulic testing system. The chemical composition of Al7075 aluminum alloy in percentage weight is listed in Table 1. The hot rolled plates have been heat-treated at 743 K for 2 h and water-quenched. Then, the plates have been heat-treated at 393 K for 24 h for aging treatment. The test specimens were initially fabricated with the tensile axis paralleling to the rolling direction from the same source block. Figure 1 shows the nominal dimension of the flat dog-bone specimen with the thickness of 2 mm. The surface of each specimen was mechanically polished to a final roughness of ~0.2 μm. These specimens were exposed to the continuous fatigue tests under the stress-control mode using the sinusoidal waveform with a constant frequency of 3 Hz and a stress ratio of 0.1. The maximum stress amplitude, *σ_max_*, was set to be 349.8 MPa, corresponding to 1.1 yield stress of the pristine specimen, which was experimentally measured prior to testing. One specimen was left undamaged and served as a reference, while the other one was cyclically loaded to fracture and the fatigue life was determined to be approximately 31,098 cycles. To produce specimens with various fatigue damage, the loading of other 11 specimens was stopped at certain cycles, which are 3000, 6000, 9000, 11,000, 13,000, 15,000, 17,000, 19,000, 21,000, 23,000 and 25,000 cycles, respectively. The test condition and fatigue life fraction of specimens are listed in Table 2.

### 3.2. Nonlinear Lamb Wave Measurements

**Figure 2:**
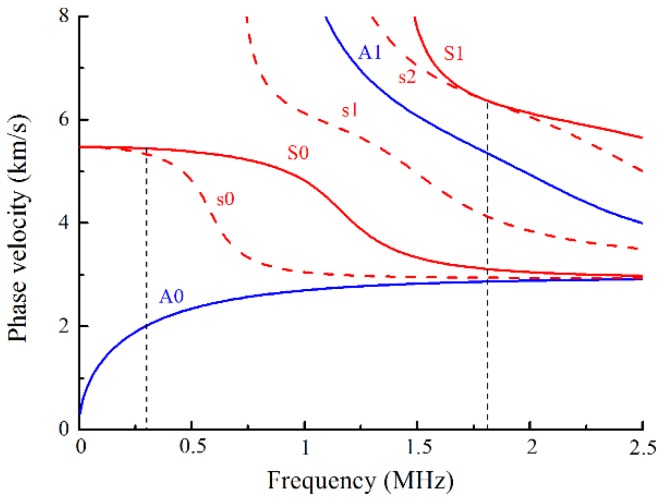
Dispersion curves of phase-velocity for Lamb waves in a 2-mm-thick aluminum alloy plate.

Measurements of nonlinear Lamb waves were performed on the specimens with various fatigue cycles. The low frequency Lamb mode pair S0-s0 at the frequency of 300 kHz was initially considered to characterize the fatigue damage in this work, as shown in Figure 2. The red solid curves represent the primary S0 and S1 Lamb modes, while the blue solid curves represent the primary A0 and A1 Lamb modes. In addition, the red dashed curves represent the second harmonic s0, s1 and s2 modes with half the frequency. These dispersion curves are numerically calculated based on the material parameters listed in Table 3, where the longitudinal and transverse wave velocities were experimentally measured on an Al7075 cube using the secondary pulse-echo method, and the third order elastic constants are cited in [4]. As illustrated in Figure 2, only the s0 mode exists in the field of second harmonics before the cut-off frequency, while this field is generally decomposed into a series of double frequency Lamb waves at a relatively high frequency. The phase-velocity changes slightly with the frequency for the Lamb mode S0 in the low frequency region. Consequently, the chosen mode pair S0-s0 satisfies the quasi phase-velocity matching condition at low frequency.

**Figure 3:**
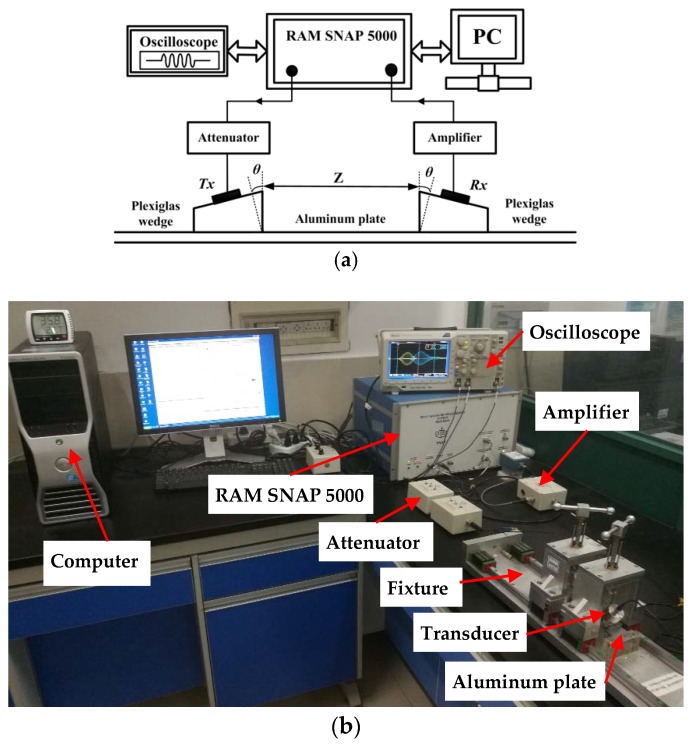
(**a**) Schematic of the experimental setup for the nonlinear ultrasonic measurements; and (**b**) photographic illustration of the experiment.

**Figure 4:**
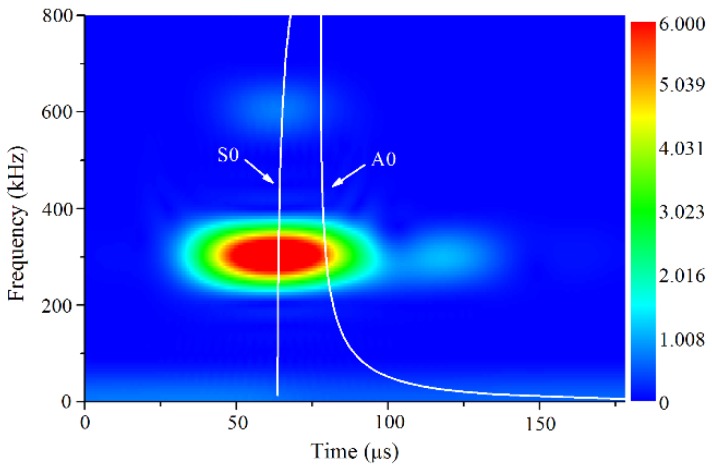
Typical frequency-time spectrogram of received signal with dispersion curves of group-velocity.

As shown in Figure 3, a 20-cycles high power Hanning windowed sinusoidal tone burst excitation voltage was generated at 300 kHz by a high-power gated amplifier (RITEC SNAP RAM-5000, RITEC Inc., Warwick, RI, USA) and fed into the transmitter, a longitudinal piezoelectric transducer with the central frequency of 300 kHz. The transducers were coupled to the plexiglas wedges with the incident angle of 29.5° determined by Snell’s law. The wedges were coupled to the specimens with light lubrication oil and aligned in parallel by a special fixture. The propagation signals were received by a longitudinal piezoelectric transducer with a central frequency of 600 kHz and sampled at 100 MHz by an oscilloscope. To enhance the signal to noise ratio, more than 512 signals were averaged to obtain the received signals. Short time Fourier transform (STFT) was performed on the received signals to acquire the amplitudes of the primary and second harmonic Lamb waves, *A*_1_ and *A*_2_, as illustrated in Figure 4. Several attempts were performed to confirm the STFT parameters for guaranteeing both the frequency and time resolutions. Note that the time delay in the wedges was considered for the dispersion curves of group-velocity. The relative acoustic nonlinearity parameter was then calculated as *A*_2_/*A*_1_^2^, which is proportional to the absolute acoustic nonlinearity parameter and can effectively characterize the evolution of material degradation.

The measurement system was initially calibrated on the pristine specimen with an increasing input voltage. The nonlinearity from the experimental equipment was eliminated for the linear increase of the second harmonic amplitude *A*_2_ with the square of the fundamental amplitude *A*_1_^2^. The accumulation of ultrasonic nonlinearity was then investigated for the Lamb mode pair S0-s0. Nonlinear ultrasonic measurements were conducted on the intact specimen at various propagation distances between the wedges from 50 mm to 170 mm with an interval of 10 mm by moving the receiving wedge transducer away from the transmitting wedge transducer. Nonlinear ultrasonic measurements were repeated five times at each propagation distance by completely removing and then reattaching the wedge transducer assembly to the plate.

Then, the fatigue damage was experimentally evaluated using the demonstrated low frequency Lamb mode pair S0-s0 at 300 kHz. Nonlinear ultrasonic measurements were performed on the specimens with various fatigue life fraction. The propagation distance between the wedges was constantly set to be 60 mm, which is an appropriate measurement distance within half the oscillation spatial period $z_{n}$, considering the length of the wedges and the gauge length of the specimens shown in Figure 1. To characterize the sensitivity of Lamb mode pairs to the fatigue damage, nonlinear ultrasonic measurements were further performed on the specimens at the same propagation distance using the Lamb mode pair S1-s2 with exact phase-velocity matching, as shown in Figure 2, since this mode pair was generally used to evaluate the material damage in previous researches. To generate the primary S1 mode, a narrowband longitudinal piezoelectric transducer (central frequency 2.25 MHz) was used to excite the tone bursts at 1.81 MHz into the plexiglas wedges with the incident angle of 25°. The signals were received by a broadband longitudinal piezoelectric transducer with a central frequency of 3.50 MHz. The other experimental parameters were identical to those used for mode pair S0-s0. To further investigate the effect of the excited frequency on the fatigue damage evaluation for mode pair S0-s0, the specimens were detected in the frequency range from 300 kHz to 400 kHz with an interval of 50 kHz, where the quasi phase-velocity matching condition is satisfied. All the measurement setup to generate and detect nonlinear Lamb waves was maintained constant, with the exception of the excited frequency.

## 4. Simulation Deployments

**Figure 5:**
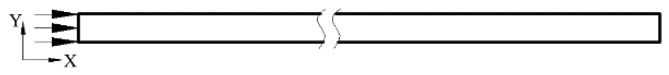
Schematic diagram of a two-dimensional FE model.

Finite element simulations of nonlinear Lamb waves propagation in a 2-mm-thick aluminum alloy plate were performed to demonstrate the cumulative generation of second harmonics and the evaluation of fatigue damage for the low frequency Lamb mode pair S0-s0 using the commercial FE software, Abaqus/EXPLICIT, where the nonlinear equilibrium equation is solved in each iteration by the central difference integration. The nonlinear constitutive relationship and the Green-Lagrange strain tensor in Equations (2) and (3) were incorporated in a subroutine VUMAT using FORTRAN, a compiled imperative programing language, for an isotropic and homogenous material in order to construct both the convective and inherent nonlinearities, regardless of the material’s attenuation and dispersion. The material parameters of aluminum alloy are shown in Table 3. The two-dimensional model was established with the length of 1000 mm, which was discretized by plane strain elements CPE4R. The upper and lower surfaces were traction-free. To obtain error convergence and calculation precision, the rectangular model was meshed uniformly with elements of size 0.02 mm much smaller than the spatial resolution λ/24, where λ is the wavelength. To satisfy stability criteria [34], the time step was set to 2.626 × 10^−9^ s, much smaller than the time resolution Δd/c, where *c* is the group-velocity of the target Lamb mode. As shown in Figure 5, the primary Lamb mode S0 was excited uniformly from the left surface of the aluminum alloy plate at 300 kHz, as the in-plane displacement of the low frequency S0 mode distributes almost linearly through the thickness. The excitation signal is formulated as *U* = *U*_0_ *A*(*t*) *B*(*Y*), where *U*_0_ is the excitation displacement amplitude of 3.5 × 10^−4^ mm, corresponding to a stress amplitude of a few MPa, is typical for Lamb wave propagation in solids; *A*(*t*) is the temporal waveform of a 20-cycles Hanning-windowed sinusoidal tone burst; and *B*(*Y*) is the thickness profile of the excitation displacement. Receivers were placed at nodes located 20–600 mm away from the excitation source with an interval of 20 mm to pick up the in-plate displacements at upper surface of the plate. STFT was performed on the received signals to extract the amplitudes of the primary and second harmonic Lamb waves and calculate the relative acoustic nonlinearity parameter with the propagation distance. Consequently, the cumulative generation of second harmonics was expected to be validated with the propagation distance for the low frequency Lamb pair S0-s0.

FE simulations were further conducted to evaluate the incipient fatigue damage using the low frequency mode pair S0-s0. As reported in previous research, the acoustic nonlinearity parameter increases significantly with the accumulation of material degradation, while the acoustic linear parameter changes slightly in the incipient stage of material damage [1,24,28,35]. Since the variation of acoustic nonlinearity parameter is ascribed to both the second-order and third-order elastic constants, while the acoustic linear parameter depends on the second-order elastic constants, it can be concluded that the third-order elastic constants mainly contribute to the acoustic nonlinearity [1,24]. Consequently, the damage evolution in the initial stage of fatigue life can be essentially characterized by the increase of third-order constants. Therefore, FE simulations were performed with the increasing third-order elastic constants. As these simulations are merely supposed to qualitatively illustrate the acoustic nonlinearity with respect to the fatigue damage, the third-order elastic constants *A*, *B* and *C* are assumed to increase equally up to *αA*, *αB* and *αC* in this work, where *α* is set to be 1, 2 and 3, respectively.

## 5. Results and Discussions

### 5.1. Cumulative Generation of Second Harmonics

**Figure 6:**
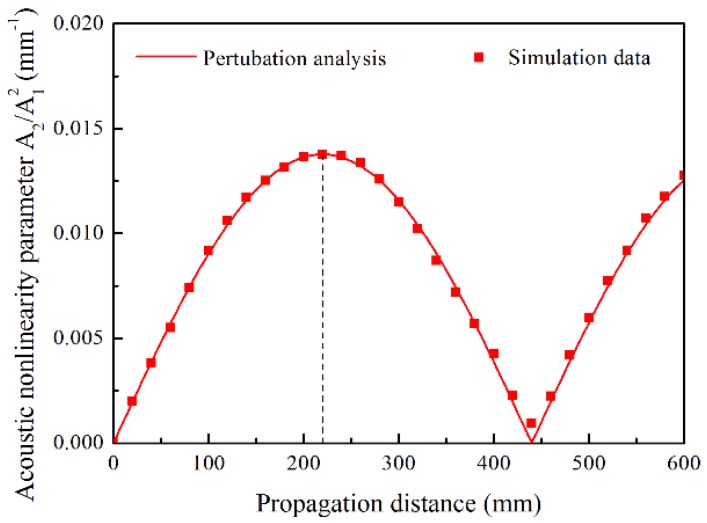
Relative acoustic nonlinearity parameter *A*_2_/*A*_1_^2^ with respect to the propagation distance. The *A*_2_/*A*_1_^2^ was acquired in perturbation analysis and simulations for mode pair S0-s0 with excitation frequency of 300 kHz.

Based on the second order perturbation approach and the FE simulations, Figure 6 shows the relative acoustic nonlinearity parameter, *A*_2_/*A*_1_^2^, with the propagation distance for the Lamb mode pair S0-s0 with the excitation frequency of 300 kHz. The *A*_2_/*A*_1_^2^ oscillates along the propagation distance in a sinusoidal behavior with an oscillation spatial period of 439.65 mm owing to the quasi phase-velocity matching condition. The oscillation amplitude and spatial period are consistent between the simulation results and the theoretical analysis, as the simulation data points locate exactly on the curve of perturbation analysis, which was theoretically acquired by Equations (1) and (2). The *A*_2_/*A*_1_^2^ grows cumulatively with the propagation distance within half the oscillation spatial period, $z_{n}$. Note that $z_{n}$ could be calculated using Equation (3), which is inversely proportional to the deviation of the phase velocities between the primary and second harmonic Lamb waves.

**Figure 7:**
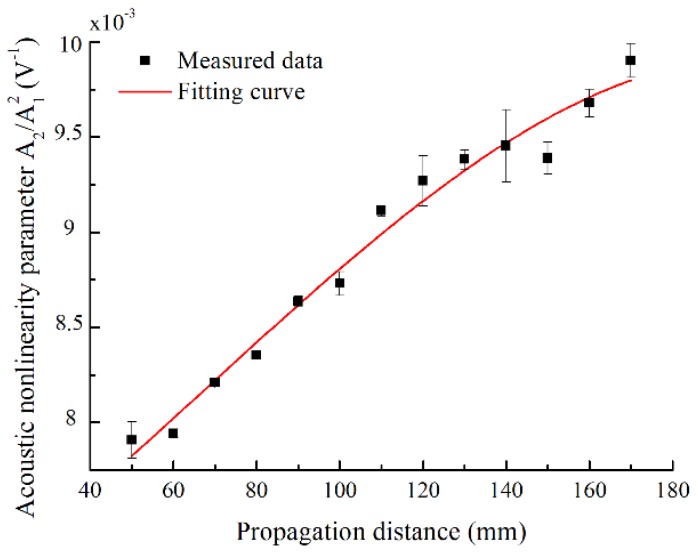
Relative acoustic nonlinearity parameter *A*_2_/*A*_1_^2^ with respect to the propagation distance. The *A*_2_/*A*_1_^2^ was experimentally measured for mode pair S0-s0 with excitation frequency of 300 kHz.

Experimental measurements of the relative acoustic nonlinearity parameter are shown in Figure 7. The cumulative increase of the relative acoustic nonlinearity parameter with the propagation distance is clearly observed from 50 to 170 mm. The error bars represent a standard deviation of the five repeated measurements. Limited to the length of the intact specimen and the emitting and receiving wedges, the maximum distance between the wedges was determined to be 170 mm, less than half the oscillation spatial period. Both the simulations and experimental measurements are consistent on the cumulative generation of second harmonic S0 mode within half the oscillation spatial period $z_{n}$, although the electrical and displacement signals are respectively received in experiments and simulations, which may contribute to the deviation of slope ratio. Consequently, the mode pair S0-s0 at low frequency may be used to effectively assess the material nonlinearity in a certain distance shorter than $z_{n}$.

### 5.2. Fatigue Damage Evaluation

**Figure 8:**
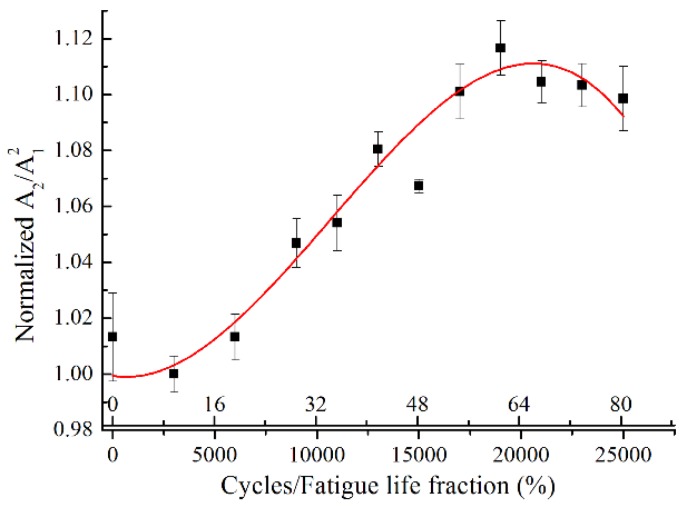
Normalized relative acoustic nonlinearity parameter *A*_2_/*A*_1_^2^ with respect to the fatigue cycles for mode pair S0-s0 with excitation frequency of 300 kHz.

Figure 8 shows the variation of the normalized relative acoustic nonlinearity parameter, *A*_2_/*A*_1_^2^, as a function of the fatigue cycles and the fatigue life fraction. For an easy comparison, the *A*_2_/*A*_1_^2^ were normalized with respect to their minimum value on the specimen with 3000 fatigue cycles. A mountain shape curve between the normalized *A*_2_/*A*_1_^2^ and the fatigue life is observed. The normalized *A*_2_/*A*_1_^2^ increases significantly with cyclic loading in the early stage of fatigue life. After about 0.65 fatigue life consumed, a peak value of normalized *A*_2_/*A*_1_^2^ is reached, which grows to nearly 112%. After this peak point, a slight decrease of normalized *A*_2_/*A*_1_^2^ is observed with the fatigue life. The error bars of the standard deviation are determined by repeating the measurements five times, which may be ascribed to the coupling conditions. The slight deviation of individual measured data from the fitting curve may be attributed to the stress control in the servohydraulic testing system, as its load capacity is relatively large for the load level in this work. Similar observation was reported for the Lamb mode pairs with the exact phase-velocity matching [32]. Consequently, the low frequency mode pair S0-s0 is found to be effective to quantitatively evaluate the evolution of the fatigue damages.

**Figure 9:**
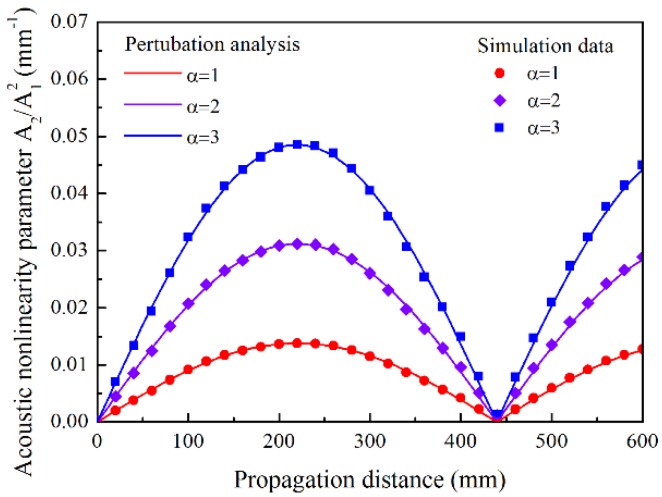
Relative acoustic nonlinearity parameter *A*_2_/*A*_1_^2^ versus propagation distance with increasing third-order elastic constants up to *αA*, *αB* and *αC* for mode pair S0-s0 with excitation frequency of 300 kHz. *α* is the scale factor.

From the point of macroscopic view, the material degradation contributes to the increase of the third-order elastic constants in the early stage of fatigue life. According to the normal analysis [3,4,5], the increasing third-order elastic constants contribute to the increase of the power fluxes $f_{Vn}(z)$ and $f_{Sn}(z)$, resulting in the growth of the amplitude of the *n*th DFLW, $a_{n}(z)$, as illustrated in Equation (2). Figure 9 shows the cumulative growth of the *A*_2_/*A*_1_^2^ obtained in simulations with the increasing third elastic constants, *αA*, *αB* and *αC*. The growth of the *A*_2_/*A*_1_^2^ with the material degradation was numerically illustrated in the early stage of fatigue life. Although the absolute increase of the *A*_2_/*A*_1_^2^ varies with the propagation distance, the relative increase of the *A*_2_/*A*_1_^2^ with respect to the initial value (*α* = 1) keeps consistent. As the relative increase of the *A*_2_/*A*_1_^2^ is independent on the propagation distance, it may be an effective parameter to characterize the material degradation, namely, the normalized *A*_2_/*A*_1_^2^. Also the consistent oscillation spatial period was observed owing to the constant parameters in Equation (3), as the second-order elastic constants are assumed to remain unchanged in the simulations.

From the point of microscopic view, the variation of the *A*_2_/*A*_1_^2^ with respect to the fatigue damage is essentially ascribed to the microstructural evolution, such as dislocation, vacancy, dislocation cell and wall. According to previous studies [26,36], in the initial stage of fatigue life, the dislocation density increases significantly and vacancy clusters are generated due to the dislocation glide in the persistent slip bands. The dislocation-induced nonlinearity is proportional to the dislocation density based on the dislocation models [36,37], while the acoustic nonlinearity caused by the vacancies is considerably smaller than that by the dislocations [36]. Therefore, the significant increase of normalized *A*_2_/*A*_1_^2^ dominantly attributed to the dislocation density. In the last stage of fatigue life, the dislocation density remains a relatively stable level, and the dislocation cells and walls are formed, while the vacancy clusters coalesce as a precursor to the nucleation and growth of microcracks [36]. The nonlinearity induced by the microcracks is found to decrease with the width of the microcracks, which dominantly contributes to the slight decrease of normalized *A*_2_/*A*_1_^2^ [38,39].

### 5.3. Analyses of Sensitivity

**Figure 10:**
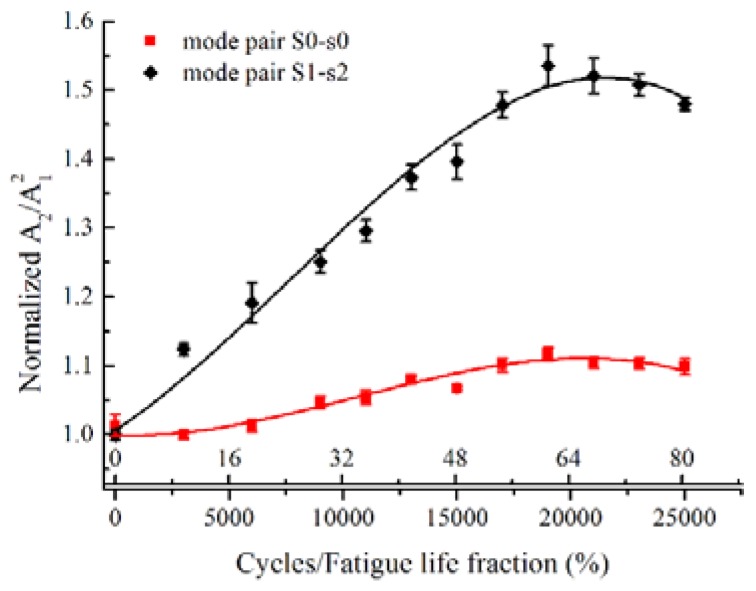
Comparison of normalized *A*_2_/*A*_1_^2^ versus fatigue cycles between mode pair S0-s0 with excitation frequency of 300 kHz and mode pair S1-s2 with excitation frequency of 1.81 MHz.

To further analyze the sensitivity of the low frequency mode pair S0-s0 to the fatigue damage, the acoustic nonlinearity of mode pair S0-s0 was compared with that of the mode pair S1-s2 with exact phase-velocity matching. Figure 10 illustrates the normalized *A*_2_/*A*_1_^2^ with the fatigue cycles and the fatigue life fraction for the mode pairs S0-s0 and S1-s2, respectively. While the mountain shape curves with peak values of normalized *A*_2_/*A*_1_^2^ at about 0.65 fatigue life are simultaneously observed for both mode pairs, the normalized *A*_2_/*A*_1_^2^ of mode pair S1-s2 is relatively larger than that of mode pair S0-s0 at each fatigue cycle. At the peak points, the normalized *A*_2_/*A*_1_^2^ reaches to nearly 155% for mode pair S1-s2 and nearly 112% for mode pair S0-s0. The mode pair S0-s0 is relatively less sensitive to the fatigue damage than the mode pair S1-s2 with exact phase-velocity matching.

The microstructural evolution during the fatigue life mainly contributes to the material nonlinearity, which could be indicated by the absolute nonlinearity parameter $\beta =\frac{8{\bar{A}}_{2}}{k^{2}z{\bar{A}}_{1}{}^{2}}f$, where *k* is the wave number, *z* is the propagation distance, ${\bar{A}}_{1}$ and ${\bar{A}}_{2}$ are the absolute displacement amplitudes of the primary and second harmonic waves, respectively, *f* is a frequency and mode dependent function for Lamb waves, and equals to 1 for longitudinal waves [40]. While the absolute nonlinearity parameter *β* is a material property dependent only on the microstructure, the relative acoustic nonlinearity parameter ${\bar{A}}_{2}/{\bar{A}}_{1}{}^{2}$ is a function of the microstructure, the mode pair and the frequency for Lamb waves. Specifically, the second harmonic generation of Lamb waves induced by the precipitate-dislocation interaction was theoretically analyzed based on the combined method of modal analysis and partial wave analysis in [41]. Therefore, the Lamb mode pairs show different sensitivities to the microstructural evolution during the fatigue life. According to the normal analysis [3,4,5], the increasing third-order elastic constants induced by the microstructural evolution contribute to a relatively larger growth rate of the amplitude of the *n*th DFLW, $a_{n}(z)$, for the mode pair S1-s2 than that for the mode pair S0-s0 at the early stage of fatigue damage, which is consistent with the experimental measurements on the sensitivity illustrated in Figure 10.

### 5.4. Effect of Frequency

**Figure 11:**
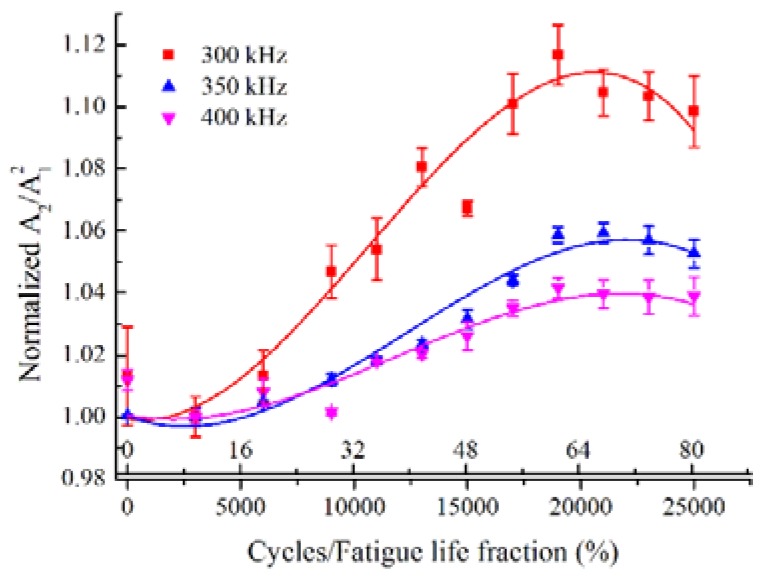
Comparison of normalized *A*_2_/*A*_1_^2^ versus fatigue cycles for S0-s0 mode pairs with excitation frequency of 300 kHz, 350 kHz and 400 kHz.

The low frequency Lamb mode pair S0-s0 may be effective to evaluate the material degradation in a range of frequency, where the quasi phase-velocity matching condition is satisfied owing to the less dispersive property. To further investigate the selection of a suitable excitation frequency to detect the fatigue damage, the comparison of acoustic nonlinearity of mode pair S0-s0 with various frequency was conducted. Figure 11 shows the normalized *A*_2_/*A*_1_^2^ with the fatigue cycles for the mode pair S0-s0 at the excited frequency of 300 kHz, 350 kHz and 400 kHz, respectively. At a lower frequency, the normalized *A*_2_/*A*_1_^2^ grows rapidly with the fatigue cycles, while the mountain shape curves with peak values of normalized *A*_2_/*A*_1_^2^ at about 0.65 fatigue life are simultaneously observed at all three excited frequencies. The mode pair S0-s0 at a relatively lower frequency are found to be more sensitive to the fatigue damage.

In addition, the lower frequency contributes to a smaller deviation of the phase velocities between the primary and second harmonic S0 mode, which leads to the increase of half the oscillation spatial period $z_{n}$, where the magnitude of second harmonics $a_{n}(z)$ grows cumulatively, as illustrated in Equation (3). Meanwhile, the envelop of the second harmonic S0 mode will be slightly stretched along the propagation path ascribed to the deviation of the group velocities between the primary and second harmonic S0 mode [32,42]. This stretch would be suppressed at a lower frequency, where a relatively smaller deviation of the group velocities occurs. Consequently, the propagation of the nonlinear Lamb wave benefits from the lower frequency. It should be noted that, based on the normal analysis [3,4,5], the $a_{n}(z)$ increases with the frequency at a specific propagation distance within $z_{n}$ for the mode pair S0-s0, which was also numerically verified in [16]. Then, the enhancement of the signal-to-noise ratio would be acquired by increasing the excited frequency. Therefore, a lower excited frequency is preferred for mode pair S0-s0 to characterize the material degradation in the premise of ensuring the signal-to-noise ratio.

## 6. Conclusions

The investigation focuses on the fatigue damage evaluation using the low frequency Lamb mode pair S0-s0 with quasi phase-velocity matching. Experimental measurements and simulations were firstly performed to validate the cumulative generation of second harmonic S0 mode at the excited frequency of 300 kHz, within half the oscillation spatial period, $z_{n}$, which was theoretically calculated using the second order perturbation approximation and modal analyses. Based on the validated mode pair S0-s0, nonlinear ultrasonic measurements were performed on the Al7075 aluminum alloy specimens with various fatigue damage, which was produced by the continuous low cycle fatigue tests. A mountain shape curve between the normalized *A*_2_/*A*_1_^2^ and the fatigue life was observed with the peak point at about 0.65 fatigue life. Compared with the mode pair S1-s2 satisfying exact phase-velocity matching condition, which was generally used to characterize the material degradation in pervious researches, the mode pair S0-s0 is found to be relatively less sensitive to the fatigue damage. Since the quasi phase-velocity matching condition was satisfied in a certain frequency range for the mode pair S0-s0 owing to the less dispersive property, the effect of excited frequency on the fatigue damage evaluation was further explored in experimental measurements. The mode pair S0-s0 at a lower excited frequency was found to be more sensitive to the fatigue damage. Consequently, the results show that the low frequency mode pair S0-s0 can be used to effectively detect the fatigue damage, and a relatively lower excited frequency is preferred in a certain frequency range.

## Figures and Tables

**Table 1:** Chemical composition of Al7075 aluminium alloy in wt %.

Si	Fe	Cu	Mn	Mg	Cr	Zn	Ti	Al
0.085	0.176	1.654	0.069	2.611	0.200	5.768	0.0438	Bal.

**Table 2:** Fatigue cycles and life fractions of the specimens.

Specimen	*σ_max_* (MPa)	*R*	*N* (cycles)	Life Fraction (%)
1	349.8	0.1	3000	9.65
2	349.8	0.1	6000	19.29
3	349.8	0.1	9000	28.94
4	349.8	0.1	11,000	35.37
5	349.8	0.1	13,000	41.80
6	349.8	0.1	15,000	48.23
7	349.8	0.1	17,000	54.67
8	349.8	0.1	19,000	61.10
9	349.8	0.1	21,000	67.53
10	349.8	0.1	23,000	73.96
11	349.8	0.1	25,000	80.39

**Table 3:** Material parameters of the used Al7075 aluminium alloy.

*ρ* (kg/m^3^)	*E* (GPa)	*v*	*σ*_0.2_ (GPa)	*c_l_* (m/s)	*c_t_* (m/s)	*A* (GPa)	*B* (GPa)	*C* (GPa)
2757.82	73.1	0.34	318	6372.70	3146.18	−351.2	−149.4	−102.8

## Abbreviations/Nomenclature

**Nomenclature**			
*A*/*B*/*C*	third order elastic constants	*k*	wave number
*A*_1_	amplitude of primary wave	*N*	fatigue cycles
*A*_2_	amplitude of second harmonic wave	*R*	stress ratio
$a_{n}(z)$	magnitude of *n*th double frequency Lamb wave	*U*_0_	excitation displacement amplitude
*A*(*t*)	temporal waveform	*w*	angular velocity
*B*(*Y*)	thickness profile	*v*	Poisson’s ratio
*c*	group velocity	*z*	propagation distance
*c_l_*	longitudinal wave velocity	*z_n_*	half the oscillation spatial period
$c_{p}^{\omega }$	phase velocity of primary wave	*α*	scale factor
$c_{p}^{2\omega }$	phase velocity of second harmonic wave	*β*	absolute nonlinearity parameter
*c_t_*	transverse wave velocity	*ρ*	density
Δ*d*	element size	*σ_max_*	maximum stress
*E*	elasticity modulus	*σ*_0.2_	yield stress
**Abbreviations**			
SHG	second harmonic generation	FE	finite element
DFLW	double frequency Lamb wave	STFT	short time Fourier transform

## References

[1] Chillara V.K., Lissenden C.J. (2016). Review of nonlinear ultrasonic guided wave nondestructive evaluation: Theory, numerics, and experiments. Opt. Eng..

[2] Jhang K.-Y. (2009). Nonlinear ultrasonic techniques for nondestructive assessment of micro damage in material: A review. Int. J. Precis. Eng. Manuf..

[3] Deng M. (2003). Analysis of second-harmonic generation of Lamb modes using a modal analysis approach. J. Appl. Phys..

[4] De Lima W.J.N., Hamilton M.F. (2003). Finite-amplitude waves in isotropic elastic plates. J. Sound Vib..

[5] De Lima W.J. (2000). Harmonic Generation in Isotropic Elastic Waveguides. Ph.D. Thesis.

[6] Zhao J., Chillara V.K., Ren B., Cho H., Qiu J., Lissenden C.J. (2016). Second harmonic generation in composites: Theoretical and numerical analyses. J. Appl. Phys..

[7] Matsuda N., Biwa S. (2011). Phase and group velocity matching for cumulative harmonic generation in Lamb waves. J. Appl. Phys..

[8] Chillara V.K., Lissenden C.J. (2012). Interaction of guided wave modes in isotropic weakly nonlinear elastic plates: Higher harmonic generation. J. Appl. Phys..

[9] Bender F.A., Kim J.-Y., Jacobs L.J., Qu J. (2013). The generation of second harmonic waves in an isotropic solid with quadratic nonlinearity under the presence of a stress-free boundary. Wave Motion.

[10] Liu M., Wang K., Lissenden C., Wang Q., Zhang Q., Long R., Su Z., Cui F. (2017). Characterizing hypervelocity impact (HVI)-induced pitting damage using active guided ultrasonic waves: From linear to nonlinear. Materials.

[11] Rauter N., Lammering R., Kühnrich T. (2016). On the detection of fatigue damage in composites by use of second harmonic guided waves. Compos. Struct..

[12] Metya A.K., Ghosh M., Parida N., Balasubramaniam K. (2015). Effect of tempering temperatures on nonlinear Lamb wave signal of modified 9Cr-1Mo steel. Mater. Charact..

[13] Lim H., Sohn H. (2017). Necessary conditions for nonlinear ultrasonic modulation generation given a localized fatigue crack in a plate-like structure. Materials.

[14] Srivastava A., Lanza di Scalea F. (2009). On the existence of antisymmetric or symmetric Lamb waves at nonlinear higher harmonics. J. Sound Vib..

[15] Sun X., Liu X., Liu Y., Hu N., Zhao Y., Ding X., Qin S., Zhang J., Zhang J., Liu F. (2017). Simulations on Monitoring and Evaluation of Plasticity-Driven Material Damage Based on Second Harmonic of S0 Mode Lamb Waves in Metallic Plates. Materials.

[16] Wan X., Tse P.W., Xu G.H., Tao T.F., Zhang Q. (2016). Analytical and numerical studies of approximate phase velocity matching based nonlinear S0 mode Lamb waves for the detection of evenly distributed microstructural changes. Smart Mater. Struct..

[17] Zuo P., Zhou Y., Fan Z. (2016). Numerical and experimental investigation of nonlinear ultrasonic Lamb waves at low frequency. Appl. Phys. Lett..

[18] Matsuda N., Biwa S. (2014). Frequency dependence of second-harmonic generation in Lamb waves. J. Nondestruct. Eval..

[19] Chillara V.K., Lissenden C.J. (2014). Nonlinear guided waves in plates: A numerical perspective. Ultrasonics.

[20] Zhu W., Xiang Y., Liu C.J., Deng M., Xuan F.Z. (2018). Symmetry properties of second harmonics generated by antisymmetric Lamb waves. J. Appl. Phys..

[21] Xiao H., Shen Y., Xiao L., Qu W., Lu Y. (2018). Damage detection in composite structures with high-damping materials using time reversal method. Nondestruct. Test. Eval..

[22] Chronopoulos D. (2018). Calculation of guided wave interaction with nonlinearities and generation of harmonics in composite structures through a wave finite element method. Compos. Struct..

[23] Gomes G.F., Mendéz Y.A.D., da Silva Lopes Alexandrino P., da Cunha S.S., Ancelotti A.C. (2018). The use of intelligent computational tools for damage detection and identification with an emphasis on composites—A review. Compos. Struct..

[24] Nagy P.B. (1998). Fatigue damage assessment by nonlinear ultrasonic materials characterization. Ultrasonics.

[25] Cantrell J.H., Yost W.T. (2001). Nonlinear ultrasonic characterization of fatigue microstructures. Int. J. Fatigue.

[26] Zhang J., Xuan F.-Z. (2014). Fatigue damage evaluation of austenitic stainless steel using nonlinear ultrasonic waves in low cycle regime. J. Appl. Phys..

[27] Kim J.-Y., Jacobs L.J., Qu J., Littles J.W. (2006). Experimental characterization of fatigue damage in a nickel-base superalloy using nonlinear ultrasonic waves. J. Acoust. Soc. Am..

[28] Jhang K.-Y., Kim K.-C. (1999). Evaluation of material degradation using nonlinear acoustic effect. Ultrasonics.

[29] Cantrell J.H. (2006). Dependence of microelastic-plastic nonlinearity of martensitic stainless steel on fatigue damage accumulation. J. Appl. Phys..

[30] Deng M., Pei J. (2007). Assessment of accumulated fatigue damage in solid plates using nonlinear Lamb wave approach. Appl. Phys. Lett..

[31] Pruell C., Kim J.-Y., Qu J., Jacobs L.J. (2009). Evaluation of fatigue damage using nonlinear guided waves. Smart Mater. Struct..

[32] Zhu W., Xiang Y., Liu C.J., Deng M., Xuan F.Z. (2018). A feasibility study on fatigue damage evaluation using nonlinear Lamb waves with group-velocity mismatching. Ultrasonics.

[33] Auld B.A. (1973). Acoustic Fields and Waves in Solids.

[34] Sewell G. (2005). The Numerical Solution of Ordinary and Partial Differential Equations.

[35] Matlack K.H., Kim J.Y., Jacobs L.J., Qu J. (2014). Review of second harmonic generation measurement techniques for material state determination in metals. J. Nondestruct. Eval..

[36] Cantrell J.H. (2004). Substructural organization, dislocation plasticity and harmonic generation in cyclically stressed wavy slip metals. Proc. R. Soc. A.

[37] Hikata A., Chick B.B., Elbaum C. (1965). Dislocation contribution to the second harmonic generation of ultrasonic waves. J. Appl. Phys..

[38] Wan X., Zhang Q., Xu G., Tse P.W. (2014). Numerical simulation of nonlinear Lamb waves used in a thin plate for detecting buried micro-cracks. Sensors.

[39] Jiao J., Meng X., He C., Wu B. (2017). Nonlinear Lamb wave-mixing technique for micro-crack detection in plates. NDT E Int..

[40] Bermes C., Kim J.-Y., Qu J., Jacobs L.J. (2008). Nonlinear Lamb waves for the detection of material nonlinearity. Mech. Syst. Signal Process..

[41] Xiang Y., Deng M., Xuan F.Z., Liu C.J. (2012). Effect of precipitate-dislocation interactions on generation of nonlinear Lamb waves in creep-damaged metallic alloys. J. Appl. Phys..

[42] Xiang Y., Zhu W., Deng M., Xuan F.Z., Liu C.J. (2016). Generation of cumulative second-harmonic ultrasonic guided waves with group velocity mismatching Numerical analysis and experimental validation. Europhys. Lett..

